# Barriers to implementation of the HIV guidelines in the IMCI algorithm among IMCI trained health workers in Zambia

**DOI:** 10.1186/1471-2431-10-93

**Published:** 2010-12-17

**Authors:** Nantalile Mugala, Wilbroad Mutale, Penny Kalesha, Elijah Sinyinza

**Affiliations:** 1 Abt Associates, Incorporation, Health Services and Systems Project (HSSP), Zambia; 2 Department of Community Medicine, University of Zambia, School of Medicine, Zambia; 3 Child Health Unit, Ministry of Health, Zambia

## Abstract

**Background:**

Zambia adopted integrated management of Childhood illnesses (IMCI) in 1995 and a number of adaptations have been made to the generic WHO/UNICEF IMCI guidelines to better conform to Zambia's health service needs. One significant adaptation is the incorporation of HIV guidelines into the IMCI algorithm. Since 2004, health workers that have undergone IMCI case management training have also received training in HIV assessment. During initial follow-up visits in 11 districts 90 health workers were assessed in 2007 to determine their adherence to the IMCI algorithm. The assessment showed that 97% of the health workers assessed did not review or mention the HIV guidelines even though they had received HIV training as part of IMCI. This study aimed to explore reasons for non-adherence to HIV guidelines in the IMCI algorithm and make recommendations on how this can be improved.

**Methods:**

Both quantitative and qualitative methods were used to collect information from eligible health workers. Forty (40) health workers were randomly selected from among those who received initial follow-up visits between March 2007 and January 2008. The health workers were selected from eight districts in four provinces of Zambia. Qualitative data was collected using Focus group discussion and key informant interviews

**Results:**

83% of the respondents said they had no difficulties in following the HIV adapted IMCI guidelines. 17% said they had difficulties. Of those who admitted having difficulties (60%) had difficulties in HIV assessment. Interesting, prompting and focus group discussions revealed that most respondents actually had difficulties in HIV assessment but could not admit it readily. Some barriers that contributed to non-adherence to the guidelines included lack of time due to inadequate staffing, lack of privacy in the health facilities and HIV related stigma from both caregivers and health workers. Frequent use of guidelines and supervision appeared to re-enforce adherence to the guidelines.

**Conclusion:**

The findings in this study suggest that training in HIV adapted IMCI guidelines is not sufficient to enable health workers to actually use their knowledge in their daily practice. Barriers may exist which prevent them from adhering to the guidelines. Addressing these barriers is critical in increasing the uptake of paediatric ART in Zambia

## Background

The number of children living with HIV has been increasing over the years. The latest estimates from UNAIDS indicate that over 370 000 children became infected with HIV in 2007 alone. Globally, the number of children younger than 15 years living with HIV increased from 1.6 million in 2001 to 2.0 million in 2007. The majority of these children are in sub Saharan Africa [1]. Zambia is among the worst affected countries with over 95,000 children already being infected with HIV through mainly mother to child transmission [2]. The adult HIV prevalence remains high at 14% with ANC prevalence being as high as 28% in some ANC Centres [3-5]. Though Zambia has scaled up the availability of ARVs to HIV positive pregnant women to help reduce mother to child transmission of HIV, many paediatric infections continue to occur [3]. It is therefore important to indentify children at risk of HIV infection and initiating antiretroviral treatment early, in order to prevent unnecessary paediatric deaths. This is crucial if Zambia is to attain child related millennium development goal of reducing under-five mortality by 2015 [6,7].

Integrated Management of Childhood Illness (IMCI) is a WHO/UNICEF strategy to improve child survival in resource poor settings [8]. Integrated Management of childhood Illnesses (IMCI) represents one of the main entry points to the health system for sick children who present at the primary care level to access HIV/ART services [8-11].

Zambia adopted IMCI for managing sick children at the primary care level in 1995. A number of adaptations have been made to the generic WHO/UNICEF IMCI guidelines to better conform to Zambia's health service needs and health system structure. These have included the revision of the malaria treatment guidelines and the incorporation of HIV guidelines into the IMCI algorithm which was completed in 2003. Since 2004, health workers who have undergone IMCI case management training have also received training in the new adaptations which have included HIV assessment, classification and referral guidelines. The training being offered at that time was 11 day IMCI course with specific time allocated to HIV. The training included both theoretical and practical sessions. Practical sessions included senarios and real patients. This is re-enforced by supervisory visits from the district and provincial IMCI teams [12,13].

The inclusion of HIV in the algorithm for IMCI was tested in South Africa where it showed that when used correctly, it can help to identify over 90% of HIV infected children [9].

As part of the IMCI case management training, health workers are expected to receive post training follow-up visits. The objectives of these visits are to:

1. Reinforce IMCI skills and help health workers transfer these skills to clinical work in facilities

2. Identify problems faced by health workers in managing cases and help solve these problems

3. Gather information on the performance of health workers and the conditions that influence performance, in order to improve the implementation of IMCI.

The visits are conducted by selected IMCI course directors in collaboration with the respective district health management team.

Between March 2007 and January 2008 Health Services and Systems Programme (HSSP) provided technical and financial support to conduct initial follow-up visits in 11 districts of Zambia. During these district visits, 90 health workers (most of whom had received training that included HIV assessment were followed up to evaluate their performance including adherence to the IMCI guidelines. This initial follow up visit indicated that most of the trained health workers (97%) did not review or mention the HIV guidelines despite receiving training. This revealed that although HIV guidelines are included in the IMCI algorithm, they are not properly utilized by health workers. This means that children eligible for HIV/ART services may not be identified in time. These findings were worrying and led to this study aimed at establishing barriers to adhering to the HIV guidelines within the IMCI algorithm and to identify ways of improving adherence to these guidelines.

## Methods

Both quantitative and qualitative methods were used to collect information from eligible health workers. The health workers included nurses, clinical officers and Environmental health technicians. Forty (40) health workers were randomly selected from among those who received initial follow-up visits between March 2007 and January 2008. The health workers were selected from eight districts in four provinces of Zambia. The selected health workers represented 44% of those health workers who received initial follow-up visits. Qualitative data was collected using Focus group discussion (Seven) and key informant interviews (Eight). Each focus group had 5-10 participants.

### Selection of survey subjects

Participants were selected based on their training in IMCI case management and having received initial follow-up visits during the stated time as well as their availability during the time of the survey. They were selected if they were screening children under-five years of age at their facility and had received IMCI training. One detailed questionnaire was administered at each site to a key informant. A key informant was one who was constantly attending to under-five children over the one month prior to the survey.

### Selection of Survey Sites

The selection of the survey sites was based on easy accessibility to both the districts and the facilities by road.

### Data Collection

Data was collected with the help of sixth year medical students under the supervision of the survey focal person and the survey epidemiologist. The data collectors received training on how to administer the questionnaire and conduct the focus group discussion. The questionnaire was pre-tested before being used in the field.

### Data Analysis

Quantitative data was entered into epidata software database. Double entry was used to ensure consistence in the entered data. Data was then exported to SPSS and STATA for analysis.

Focus group discussion and key informants' data was analyzed using open-code software for qualitative data (Umea University, Sweden)

### Ethical Issues

Permission for the study was obtained from the MoH through the directorate of public health and research and verbal consent was obtained from survey participants (health workers) prior to enrolment into the survey. Participation in the survey was voluntary and no incentives were given.

## Results

### District description and respondents demographic characteristics

Eight districts were visited. These included: Chadiza, Chipata (Eastern province), Kalulushi, Chingola (Copperbelt province), Kapiri-Mposhi, Mkushi (Central province) and Mufumbwe, Solwezi (North-Western province). Majority of respondents were from Kalulushi (20.7%) followed by Mkushi and Chadiza at 13.8% of respondents each.

**Figure 1:**
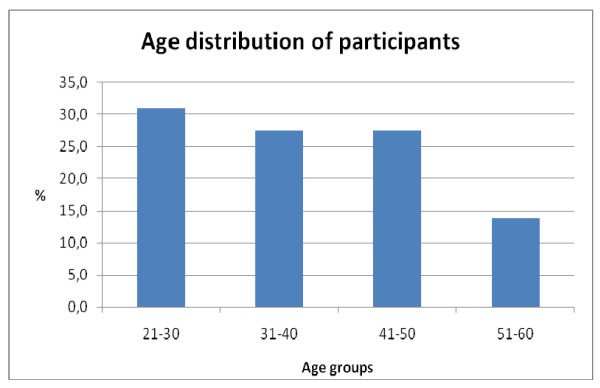
Age distribution of participants

Majority (31%) of respondents were nurses between the ages of 21 to 30 while those between 51 to 60 years were only 14% (Figure 1). The sample size consisted of more females (52%) than males (48%). All respondents were Christians by religion. (Table 1)

**Table 1:** Description of Districts visited, demographics of staff and supervisory visits

Variable	n	(%)
**Districts**		
Chadiza	4	13.8
Chipata	3	10.3
Kalulushi	6	20.7
Chingola	3	10.3
Capri Mposhi	3	10.3
Mkushi	4	13.8
Mufumbwe	3	10.3
Solwezi	3	10.3
		
Cadre*		
Nurses	12	66.7
Clinical officers	3	16.7
Environmental health technician	3	16.7
		
**Age groups**		
21-30	9	31.0
31-40	8	27.6
41-50	8	27.6
51-60	4	13.8
**Sex**		
Male	14	48.3
Female	15	51.7
		
**Religion**		
Christian	29	100.0
		
**# of supervisory visits**		
0	3	10.3
1	22	75.9
2	4	13.8
**Year of last supervisory visit**		
2005	2	6.9
2006	1	3.4
2007	24	82.8
2008	2	6.9

Note:* 11 cadres not included because data was not available

### Supervision on and Adherence to IMCI/HIV guidelines

Table 1 shows that most of the respondents recalled having received at least one supervisory visit (90%). 10% said they had not received any supervisory visit after training in IMCI. Respondents were asked whether they had any difficulties following the IMCI algorithm, 83% said they had no difficulities.17% said they had difficulties following the IMCI algorithm. Out of those who had difficulties, majority 60% (3/5) cited HIV assessment as the area where they had difficulties.

90% of the participants said they had received training in HIV guidelines (62% through the standard training and 28% through peer orientation).

When asked about conditions which could prompt them to enter the HIV box, most of them mentioned growth faltering (82%).

When asked about the adequacy of training received in IMCI, majority of the respondents agreed that the training was adequate (62%), while 31% said the training was not adequate.

Most participants had received training in HIV counseling (52%) or had attended some HIV course (54%). (See Table 2)

**Table 2:** HIV algorithm adherence and associated factors

Variable	n	%
**Do you have any area of difficulty in the algorithm:**		
Yes	5	17.2
No	24	82.8
		
**Common areas of difficulties:**		
The General danger signs	1	3.4
The main symptoms	1	3.4
Assessing for malnutrition & malaria	1	3.4
Assessing for HIV	3	10.3
Assessing for feeding	1	3.4
		
**Received training in use of HIV algorithm**		
Yes	18	62.1
No	11	37.9
		
**If no to above, any knowledge about HIV guidelines**		
Yes	8	27.6
No	3	10.3
		
**Which conditions in a child would prompt you to enter the HIV box**.		
Persistent diarrhea	19	65.5
Pneumonia	19	65.5
Growth faltering or low weight for age.	24	82.8
Chronic ear infection	8	27.6
others	19	67.9
		
**Do you find it difficult to do the HIV assessment?**		
Yes	3	10.3
No	25	86.2
		
**If yes to above, why(reason)**		
Lack of time	0	0.0
Poor staffing	0	0.0
Not comfortable to discuss HIV issues	0	0.0
Not well trained	1	3.4
others	2	6.9
		
**Reasons for not adhering to the HIV guidelines**		
Shortage of staff	23	79.3
oversight	14	48.3
Stigma of staff towards HIV	5	17.2
Stigma in the community	19	65.5
Inadequate training	16	55.2
Guidelines not user friendly	2	6.9
		
**Do you think you were adequately trained in IMCI**		
Yes	18	62.1
No	9	31.0
No response	2	6.9
		
**Trained in HIV counseling**		
yes	14	51.9
No	11	40.7
No response	2	7.4
		
**Received training in HIV**		
Yes	14	53.8
No	10	38.5
No response	2	7.7

### Key informant interview

7 out of the 8 key informants recalled having received initial follow-up supervision at least once. One respondent did not recall having received initial follow-up visit. *When asked about the benefits of the supervisory visits*, all the respondents agreed that these were helpful. One respondent said, "Yes, they helped me to get updated on important issues for example the treatment of dysentery and they also emphasised the use of guidelines. Another one said "it helped me to follow the steps outlined in the guidelines properly instead of jumping some parts"

*When asked about any areas of difficulty*, 5 out of the 8 respondents reported having problems with guidelines. One said he had difficulties with the HIV section, while 3 had difficulties with the immunisation section.

Majority denied seeing other colleagues having difficulties with any section. But 2 of the 8 respondents said they had seen colleagues having difficulties following the guidelines.

*When asked if they had received training in the use of the HIV guidelines*, six of the eight respondents said they had received specific training in HIV guidelines. Those not trained attributed this to the absence of the HIV component in their training Programme.

*On specifically being asked if they had difficulties with HIV guidelines*; Two respondents acknowledged having difficulties in following HIV guidelines, while the rest denied having any difficulties.

In explaining reasons for not following the HIV guidelines, one respondent said" The IMCI guidelines are not well arranged and so it is not easy to access the HIV box". Another said "the booklet takes too long especially when you have too many patients"

*The respondents were also asked about the role of stigma in adherence to the HIV guidelines*. Three of the eight respondents said stigma had a role in hindering adherence to the HIV guidelines. Reasons cited for this included:

i. Caretakers who are not real parents to a child were not open to discussing HIV.

ii. Failure by HIV positive mothers to disclose their HIV status to their spouses. This made it difficulty for the mother to discuss the status of the baby when the spouse is not aware even of the HIV status of the wife.

### Focus group discussion

*The discussants were asked if they had any problems following the IMCI algorithm*. Most of them said they had no difficulty in following these guidelines and few said they had difficulties. The responses varied from having completely no difficulties to those who gave conditions. The phrase "have no difficulties" was mentioned 17 times by discussants. "So far I have no difficulties and everything is clear" and another one said "this book is easy to follow". Other discussants used conditional sentences such as: "I have no difficulties if there are fewer patients" or "if the patient is stable". *When asked specifically what they think about HIV assessment whether it is easy or difficult*. Many discussants replied that it was easy to follow. "I have no problems since I have undergone training in counselling". Some conditions appeared to be important in determining whether the HIV guidelines were easy to use or not. For example issues of frequency in using the guidelines or practice were mentioned. "it is ok when you use it frequently some things will stick." Another one said "For those of us doing it practically we no longer have difficulties.

Discussants gave reasons why health workers were not adhering to the HIV guidelines. Some reasons where from personal experience while other reasons where observed or perceived. The element of time was mentioned 14 times during the discussions. IMCI guidelines were said to be time consuming. This was closely related to busy schedules, high number of patients or poor staffing. In relation to time one discussant said "due to pressure of work and shortage of staff, it is difficult to spend a long time on each child. Echoing similar sentiments, another discussant said "the booklet is time consuming and if you keep on referring to it, you will be perceived as being incompetent by the caretaker". Another respondent added" I think in fact here in rural areas you attend to both adults and children, if there are a lot of patients in the morning, it becomes very difficult to stick to the guidelines."

Other reasons for not adhering to HIV guidelines included the negative attitudes among some health workers and mothers or caregivers presenting with other symptoms rather than those that may point to HIV. The fact that HIV was not an emergence made some health workers concentrate on other emergences, hence not paying attention to the HIV section. According to some discussants, the HIV guidelines had too much detail, hence it was confusing. Some blamed lack of training in HIV counselling. Others cited lack of privacy in the screening rooms or lack of space in out-patient departments. Stigma both in the community and among members of staff was also mentioned as a contributing factor to non adherence to HIV guidelines.

The lack of feedback after referring patients or lack of transport money to refer patients to counselling centres were cited as other reasons discouraging some health workers from talking about HIV to mothers. Health workers felt helpless as they felt they where not helping clients fully. They could not confirm whether the mother actually went for HIV testing or not after being referred.

All discussants appreciated the role of supervisory visits. They said that they had a positive effect on those visited as reflected in the following responses. "We tend to forget- but my supervisor during my follow up visit reminded me... it has a good effect". "I had stopped using the algorithm but when I was visited... I was forced to start using the IMCI algorithm...It really helps us to follow the guidelines properly it encourages us to do what you are expected to do instead of short cuts." Referring to supervisors another respondent added, "It's actually very pleasing and you feel you are being supported, yes supervision can help."

## Discussion

These results point to the fact that training in HIV and provision of IMCI guidelines is not sufficient to enable health workers to actually use their knowledge in their daily practice. The role of supervision, staff shortages and stigma in adhering to guidelines warrants further research [10]. It was also interesting to note that when individual respondents were asked whether they had any difficulties following the IMCI algorithm, 83% said they had no difficulties in following HIV assessment guidelines. However closer examination revealed that many health workers had difficulties in HIV assessment as evidence by wrong answers given when asked to indicate conditions necessary to enter the HIV box and most admitted this during focus group discussions. This finding highlights the importance of using multiple methods in assessing health workers during supervision.

The adherence to HIV guidelines seem to correlate well with frequency of use of the guidelines. Our study established that those who used the booklet frequently were able to master the key elements of the guidelines compared to those who used the guidelines infrequently. The findings of this survey suggest a lack of constant use of the guidelines contributed to health workers failure to adhere to the HIV guideline which was cited during the initial follow-up visits. During the focus group discussion, health workers mentioned that the HIV guidelines were easy to remember if one referred to them frequently. The health workers responded more freely when they were asked to comment on their perception on why they or other health workers may not adhere to HIV guidelines. This was especially noted in the focus group discussion where some of the health workers went ahead to give personal experiences.

Several issues were highlighted in this survey which suggests that including HIV in the IMCI algorithm and training the health workers may not necessarily translate into effective usage of the guidelines at the health centres. Among these was the issue of case load and the perception that HIV was not an emergency and therefore where a health worker had to make time, it appeared logical to concentrate on the symptoms that the child presented with and not go into the details of assessing for HIV even when there was an indication. The HIV guidelines were perceived as being very detailed and time consuming. This then suggests that the inadequate staffing levels that exist at the primary level of care needs to be addressed if the health worker is expected to dedicate quality time to every child [14]. The lack of private screening rooms in most of the health centres coupled with stigma in the communities was cited as an important reason why some of the health workers were uncomfortable to discuss HIV issues. Stigma among the health workers did not seem to be a major problem although this is difficult to ascertain. Training in counselling skills was noted to be an added advantage.

Although only about 7% of the health workers felt that the guidelines were not user friendly, 55% felt that the training in the HIV guidelines was inadequate. Furthermore during the focus group discussion it was noted that the workers were uncomfortable with being seen to look through the chart booklet in front of the caretaker. This, they felt may be mistaken to mean that the health worker is incompetent and hence may lose the confidence of the clients.

All the health workers appreciated supervision as a key element to ensure adherence to HIV guidelines. In a study done by Chitembo and others looking at the impact of health centre based supportive supervision on performance of health workers trained in IMCI, it was noted that performance was better in the intervention sites where there was constant supervision. However the same study noted that performance of the health worker may be affected negatively due to high staff attrition leading to increased work load. The guidelines on effective implementation of the IMCI guidelines have highlighted the importance of supervision and that training alone is not enough [9].

## Conclusions

The survey revealed that although health workers may not necessarily find the HIV guidelines difficult to follow, there are barriers which prevent them from adhering to the guidelines. Some of these barriers include inadequate staffing levels at some of the health facilities, prevailing stigma among both the health workers and the community, infrequent supervision of the health workers and lack of simplified wall charts. Training in counselling skills and allocation of more time to the HIV component during the IMCI training may be an added advantage.

Addressing these barriers is critical in achieving the vision of MoH which is to increase the up take of ART by the paediatric population, through the provision of free ART services and strengthening the necessary linkages between related programmes.

## Competing interests

The authors declare that they have no competing interest

## Authors' contributions

All authors contributed to the paper. NM, PK and ES, were involved in initiating the survey. WM led in the data collection and analysis. All the authors participated in drafting the paper and reviewed the final draft of the manuscript.

## Pre-publication history

The pre-publication history for this paper can be accessed here:

http://www.biomedcentral.com/1471-2431/10/93/prepub
